# Apoptosis, Cell Cycle and Differentiation Effects of Propolis Extracted in Olive Oil on HL‐60 AML Cells: A Flow Cytometric Analysis

**DOI:** 10.1155/ancp/7215006

**Published:** 2025-12-22

**Authors:** Hamide Dogan, A. Ata Ozcimen, Erkan Alabas, Sibel Silici, Yasemin Saygideger, Birgul Ozdemir

**Affiliations:** ^1^ Department of Translational Medicine, Institute of Health Sciences, Cukurova University, Adana, Türkiye, cu.edu.tr; ^2^ Department of Biology, Faculty of Sciences, Mersin University, Mersin, Türkiye, mersin.edu.tr; ^3^ Agricultural Biotechnology Department, Seyrani Faculty of Agriculture, Erciyes University, Kayseri, Türkiye, erciyes.edu.tr; ^4^ Erciyes Technopark, Nutral Therapy Co., Kayseri, Türkiye; ^5^ Department of Pulmonary Diseases, Faculty of Medicine, Çukurova University, Adana, Türkiye, cu.edu.tr

**Keywords:** apoptosis, differentiation, DNA cell cycle, olive oil extract of propolis

## Abstract

In recent years, researchers have focused on the drug capacity of natural products and their components with low side effects in the treatment of leukemia. This study aims to elucidate the antileukemic mechanism of action of olive oil extract of propolis (OEP) together with methylprednisolone (MP) in HL‐60 cells, an acute myeloid leukemia cell line. The WST‐8 kit was used to test the cytotoxic and proliferative effects of OEP on HL‐60 cells. Flow cytometry was additionally applied for determining apoptotic status, cell cycle, and differentiation levels (CD11b, CD14, and CD68). The IC_50_ concentration of OEP in HL‐60 cells was determined to be 0.5 *μ* L/mL after a 24 h period. OEP effectively arrested the cell cycle at the G_2_/M phase after 24 h, and its addition of MP strengthened this effect. After 48 h, OEP significantly inhibited the cell cycle at the G_2_/M phase. The combination of OEP with MP led to significantly greater numbers of cells arrested in the G_0_/G_1_ phase. OEP alone has shown significant efficacy in encouraging apoptosis; however, its combination with MP provided considerably more beneficial effects. The OEP performed efficiently at CD11 levels for the first 24 h. Due to its impact on apoptosis, cell cycle arrest, and differentiation, OEP may be a natural product that merits additional preclinical investigation in leukemia models. The combined effects of OEP and MP on stopping the cell cycle provide a possible way to treat leukemia. Additional research is required to investigate the processes underlying the improved efficacy of the combo‐therapy.

## 1. Introduction

The treatment of diseases with natural products dates back to ancient times. Recently, studies have increased to elucidate the mechanism of action of herbal and apitherapeutic products that have low side effects and cause minimal damage to healthy tissues [1]. Propolis, a natural product formed by substances collected from leaves, bark, buds, and shoots of plants by honeybees (*Apis mellifera* L.) and modified by bees’ saliva enzymes, has become the focus of many biological efficacy studies [2]. In the last 40 years, many researchers have focused on the chemical composition, biological activity, pharmacological, and therapeutic properties of propolis. The first known studies on this subject were made by Ghisalberti in 1979 [3]. However, new information about the biological activity and chemical structure of propolis was obtained ~20 years after these studies [4–6].

Although the plant varieties in which propolis is collected intensively vary according to the region and season, especially pine and poplar species, as well as horse chestnut, willow, alder, fir, plum, elm, oak, and ash are important sources of propolis [7]. Poplar (also located in Turkey) and Baccharis (Brazilian) species draw attention in most cancer research with propolis [8, 9]. Propolis is used in medicine, apitherapy, and biocosmetics for its antibacterial, antiviral, antifungal, antiparasitic, antioxidant, antiulcer, immunostimulatory, liver protective, and food preservative effects [10, 11]. The main compounds responsible for the biological activities of propolis are flavonoids, aromatic acids, phenolic acids, and esters [8, 12]. Compounds such as apigenin, genistein, p‐coumaric acid, pinosembrin, pinobanksin, galangin, caffeic acid phenyl ester, benzyl ester, sakuranetin, ferulic acid, quercetin, and luteolin are largely responsible for the antimicrobial, antifungal, and antiviral activity of propolis [13, 14]. The ratio of these compounds can vary according to the biogeography, season, and bee colony where propolis is collected; their biological activities can also show variability [15, 16]. To be used in biological analysis, propolis should be extracted with compounds such as ethanol, methanol, and water [17, 18]. Numerous studies highlight the use of propolis and its active ingredients in cancer therapy, showing their potential in developing new anticancer agents [19–21]. In many in vivo and in vitro studies, propolis has antitumor properties and can enhance the effects of chemotherapy, reducing or eliminating treatment side effects [22, 23].

The chemopreventive activity of propolis is due to its ability to inhibit DNA synthesis in tumor cells, induce apoptosis, and activate macrophages that regulate the functions of B‐, T‐, and NK‐ cells [24, 25]. The flavonoids in propolis are largely responsible for its antitumor effect, and the synergy between these substances is very important [26, 27]. The use of propolis as a dietary supplement on a regular schedule may help protect against cancers caused by mutations [28].

Blood cells originate from hematopoietic stem cells with high differentiation capacity. These cells, which can respond to all hormone receptors, form mature blood cells due to changes in pituitary hormone levels [29, 30]. Leukemia, known as blood cancer, is a malignant disease originating from the hematopoietic system and defined as a clonal and neoplastic blood disease. Transcriptional inactivation of many genes working in the pathways that regulate cell proliferation, differentiation, and apoptosis can cause leukemia [31].

Glucocorticoids (GCs) are used in numerous medical applications, primarily in autoimmune diseases and cancer treatments, to reduce inflammation and support immunological therapy. Methylprednisolone (MP), a member of the potent gC and steroid hormone family, is used in combination treatment protocols with agents such as all‐trans retinoic acid (ATRA) and arsenic trioxide in various types of leukemia. MP interacts both structurally and functionally with steroid hormones and other nuclear‐binding ligands such as vitamin D3 and retinoic acid via intracellular glucocorticoid receptors (GRs) to inhibit the transcription of inflammatory genes and initiate the transcription of antiinflammatory genes.

This study aimed to determine the antileukemic effects of olive oil extracts of propolis samples obtained from Turkey on AML cell line HL‐60 cells. For this purpose, it was focused on the cytotoxic, apoptotic, and antiproliferative interactions of MP and propolis, which has a high antioxidant capacity.

## 2. Materials and Methods

### 2.1. Reagents

The cell line, kits and chemicals used in this study are as follows: acute myeloblastic cell line; HL‐60 cells (ATCC, Manassas, Virginia, USA), Roswell Park Memorial Institute (RPMI‐1640) Medium (Hyclone Laboratories Inc., Cytiva), FBS (Hyclone Laboratories Inc., Cytiva), Annexin V Apoptosis Dedection Kit APC (Biolegend, San Diego, CA) USA), PI (Invitrogen, Thermo Fischer Scientific, Waltham, Massachusetts, USA), PBS (Gibco), Penicillin Streptomycin (Sigma Aldrich, USA), Amphotericin B Solution (Hyclone), MP (MN‐Mustafa Nevzat), WST‐ 8 Kit (CCK‐8; Sigma Aldrich, USA), DPBS (Sigma Aldrich, USA).

### 2.2. Olive Oil Extract of Propolis (OEP)

The OEP obtained from Erciyes University Technopark (Nutral Therapy Company, TR) is a standardized product, ensuring it meets the required experimental properties. The chemical composition and phenolic compounds of OEP are given in Table 1 and, respectively [32].

**Table 1:** Chemical composition of OEP.

Energy	875 kcal/100 g	Oil (acid hydrolysis)	98.42 g/100 g
Moisture	0.18 g/100 g	Omega 3	5.87 g/100 g
Ash	0.13 g/100 g	Omega 6	0.80 g/100 g
Protein	N.D.	Total phenolic content	1521.93 mg GAE/100 g
Carbohydrate	1.27 g/100 g	Antioxidant activity	22.75 mg AAE/g

Abbreviations: N.D., not determined; OEP, olive oil extract of propolis.

### 2.3. Cell Culture Method

HL‐60 cells were incubated in RPMI 1640 medium containing 10% fetal bovine serum, at 37°C, 5% CO_2_, and 95% humidity. The proliferative and cytotoxic dose was determined in HL‐60 cells exposed to different doses of OEP.

### 2.4. Proliferation and Cytotoxicity

Proliferation in HL‐60 cells treated with OEP for 24, 48, 72, and 96 h was determined using the WST‐8 kit. Different concentrations of OEP (0.1, 1, 5, and 10 µL/mL) in 100 µL culture medium in a 96‐welll plate were applied to the cells and incubated at 37°C. The WST‐8 kit (10 *μ* L) was added to each well, and spectrophotometric reading was performed at 450 nm wavelength in ELISA microplate reader after 2–4 h. According to the obtained OD values, IC_50_ values determined with the GraphPad Prism 8.4.3 program were applied to the cells. Cell viability data were analyzed using one‐way ANOVA followed by Dunnett’s multiple comparison test. Statistical significance was set at *p*  < 0.05.

### 2.5. Apoptosis

The early and late apoptotic cells were analyzed using Annexin V‐APC (5 µL) and PI (5 µL) dyes according to the BD Annexin V‐APC kit procedure (*n*:3). The apoptotic effect in HL‐60 cells treated with OEP, MP, and their combinations was analyzed with the BD FACSAria III flow cytometer. The results were evaluated as “primary apoptotic cells” staining with Annexin V‐APC, “secondary apoptotic and necrotic cells” staining with Annexin V‐APC and PI, “debris (fragmented) cells” staining with PI, and “live cells” not staining with Annexin V and PI. The BD FACSAria III was calibrated using BD Cytometer Setup and Tracking (CS&T) beads to verify instrument performance and ensure measurement accuracy. Control samples labeled with APC or PI were used to adjust fluorescence compensation, which minimized spectral overlap by adjusting the detector voltage.

### 2.6. DNA Cell Cycle

Flow cytometric DNA cell cycle analysis in AML cell line HL‐60 cells treated with OEP, MP, and combinations was carried out using the PI staining protocol (n:3). Treated and untreated cells were collected after 24 and 48 h of incubation and washed with PBS and fixed on vortex by adding 70% cold ethanol dropwise. The cells were incubated at 4°C for 30 min, washed twice with PBS, and centrifuged at 850*g*. RNase (50 µL, 100 µg/mL stock solution) and PI (200 µL, 50 µg/mL stock solution) were added to the cells. After all procedures, the number of cells in G_0_/G_1_, S, and G_2_/M phases in HL‐60 cells was determined on the BD FACS Aria III device. Prior to data acquisition, daily quality control and performance verification of the BD FACSAria III were performed using BD CS&T beads. Single‐stained PI control was used to adjust voltage and gating. Since only PI was applied, no fluorescence compensation was required. The obtained flow cytometric data were analyzed in the BD Mod Fit Analysis program.

### 2.7. Differentiation

Time/dose dependent MP, OEP and their combinations were administered alone and in combination for 24, 48, and 72 h to acute myeloblastic leukemia cells, and then differentiation was determined by flow cytometric measurement of granulocytic cell surface antigens (CD11b, CD14, and CD68) (Biolegend, San Diego, CA, USA). In order to determine the cell antigens (CD11b, CD14 and CD68), HL‐60 cells were stained with APC antihuman CD11b (5 µL), PE antihuman CD14 (5 µL) and FITC antihuman CD68 (5 µL) using with APC Mouse IgG1, PE Mouse IgG1 and FITC Mouse IgG2b isotype controls (Biolegend, San Diego, CA. USA). Then they were incubated at + 4°C in the dark for 30 min. Stained cells were diluted with 1 mL of PBS and analyzed in flow cytometry using the FACS‐Diva software program. A minimum of 10,000 events were acquired per sample to ensure statistical robustness. Before data acquisition, the performance and calibration of the BD FACS Diva were verified using BD CS&T beads to ensure instrument accuracy and consistency. Voltage settings and fluorescence compensation for APC, PE, and FITC channels were established in BD FACS Diva software using single‐stained controls to correct spectral overlap and ensure precise signal separation. Gates for positive cells were set using isotype controls (APC Mouse IgG1 for CD11b, PE Mouse IgG1 for CD14, and FITC Mouse IgG2b for CD68) and kept the same across all treatment groups and time points. Non‐parametric statistical comparisons (Kruskal–Wallis) were performed to compare the percentage of marker‐positive cells between treatment groups and controls.

### 2.8. Evaluation of Data

Statistical analysis of the data obtained by applying time/dose‐dependent MP and OEP alone and in combination to HL‐60 cells was performed using one‐way ANOVA followed by Tukey post hoc test in GraphPad Prism 8. The limit of statistical significance was set at 0.05.

## 3. Results

### 3.1. The Cytotoxicity Analysis in HL‐60 Cells Treated With Olive OEP

**Figure 1:**
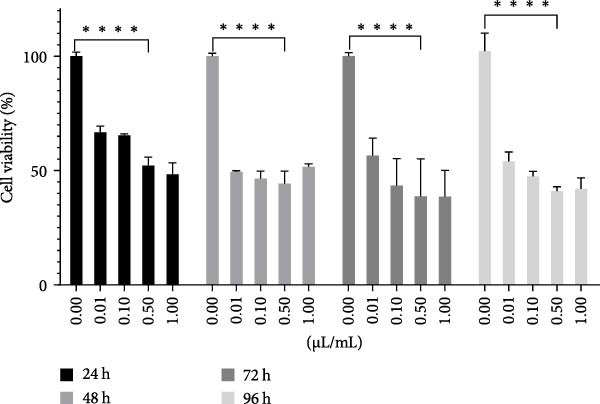
Effect of different concentrations of OEP on the viability of HL‐60 cells at 24, 48, 72, and 96 h. Data are presented as mean ± SD (*n* = 8). Statistical significance was determined using one‐way ANOVA followed by Dunnett’s post‐hoc test.  ^∗^ *p* < 0.05,  ^∗∗^ *p* < 0.01,  ^∗∗∗^ *p* < 0.001,  ^∗∗∗∗^ *p* < 0.0001 compared to control.

In order to determine the proliferative and cytotoxic dose of OEP, a standardized product, it was applied to HL‐60 cells for 24, 48, 72, and 96 h (n:8). Spectrophotometric measurements were performed on the ELISA microplate reader using the WST‐8 kit, which gave sensitive results after the application. The data obtained was analyzed with the GraphPad Prism 8.4.3 program (Figure 1). In HL‐60 cells, 0.01, 0.1, 0.5, and 10 µL/mL doses of OEP were applied. The IC_50_ dose was determined as 0.5 µL/mL.

### 3.2. The Flow Cytometric Determination of the Effect of OEP on Apoptosis in HL‐60 Cells

**Figure 2:**
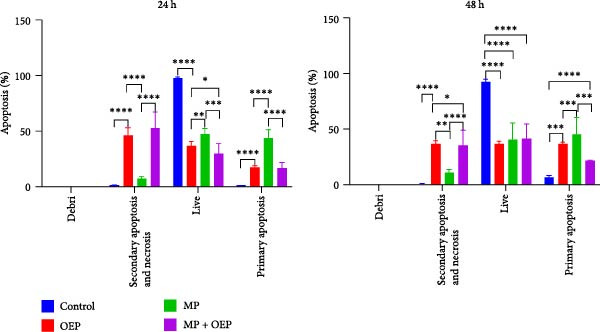
Apoptosis data obtained flow cytometrically in HL‐60 cells (n:3). The results were evaluated as follows: “primary apoptotic cells” staining with Annexin V‐APC; “secondary apoptotic and necrotic cells” staining with Annexin V‐APC and PI; “debris (fragmented) cells” staining with PI; “live cells” not staining with Annexin V or PI. The values given in the figure are expressed as mean ± standard error values. Statistical significance values of all groups compared to the control group using one‐way ANOVA with Tukey’s post hoc test are as follows: *p* < 0.05 ( ^∗^), *p* < 0.01 ( ^∗∗^), *p* < 0.001 ( ^∗∗∗^), *p* < 0.0001 ( ^∗∗∗∗^).

The ability of MP and OEP used in supportive therapy in AML patients to induce apoptosis in HL‐60 cells was analyzed by flow cytometry after application to HL‐60 cells alone or in combination. For each hour, groups were administered as follows: (1) Control, (2) OEP (0.5 µL/mL), (3) MP (5 x 10^−4^ M), and (4) MP (5 x 10^−4^ M) + OEP (0.5 µL/mL). Using Annexin V and PI dye, viable, dead, and apoptotic cells were evaluated according to the percentage (%) in the Q1, Q2, Q3, and Q4 quadrants formed in flow cytometry (Figure 2) (*n* = 3).

Apoptosis status is compared with the control groups by flow cytometric analysis of AML cell line HL‐60 cells to which MP, OEP, and their combination are applied. The 24 h flow cytometry data are as follows: Apoptosis was significantly induced in the cells to which OEP was applied (*p*  < 0.0001). In addition, although the primary apoptosis level was lower than the control and other treatment groups (*p*  < 0.0001), the secondary apoptosis level (46.18 ± 2.79) was significantly higher (*p*  < 0.0001). In the MP‐treated group, cells were significantly directed to apoptosis (44 ± 3%) (*p*  < 0.0001), and necrosis was not observed. It was observed that the secondary apoptosis level (53.1 ± 6) was the highest in the cells treated with MP + OEP (*p*  < 0.0001). Total apoptotic status at 24 h is 51% in the MP alone group and 63.51% in the OEP alone group, while it is 69.62% in the combination group. The combined administration of MP OEP resulted in a significantly higher percentage of late apoptosis. While the number of viable cells is quite low in this group compared to the control and other group cells, the secondary apoptosis level is high.

When compared with the control group at 48 h, the primary apoptosis level increased in all groups. However, since the primary apoptotic levels of the MP, OEP, and MP + OEP groups were 46 ± 6%, 32.35 ± 3% and 21.65 ± 0%, respectively, an antagonistic effect was observed in the combination group. When the secondary apoptotic cell percentages (OEP: 32.13 ± 3%, MP: 11 ± 1, and MP + OEP: 35.9 ± 6) were examined, the rate was significantly higher in the combination group compared to the MP group, and showed a notable combination effect compared to OEP alone. The total apoptotic cell percentages of MP, OEP, and MP + OEP at 48 h were 64.48%, 57%, and 57.55%, respectively, and it was observed that the combination application reduced the effect of OEP.

### 3.3. Flow Cytometric Determination of the Effect of OEP and MP on DNA Cell Cycle in HL‐60 Cells

**Figure 3:**
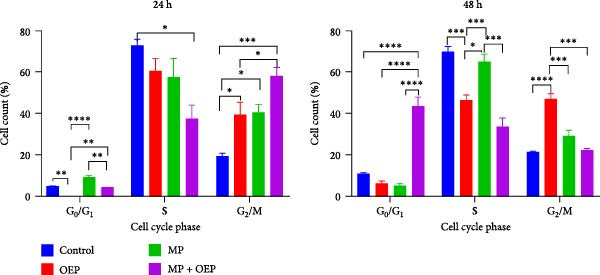
Flow cytometric analysis of the effect of OEP and MP, both individually and in combination, on the distribution of HL‐60 cells across the cell cycle phases at 24 and 48 h. The data represent the percentage of cells in the G_0_/G_1_, S, and G_2_/M phases of the cell cycle (mean ± SD of three independent experiments). Statistical differences among the groups were determined using one‐way ANOVA followed by Tukey’s post hoc test. Statistical significance levels are indicated as follows: *p* < 0.05 ( ^∗^), *p* < 0.01 ( ^∗∗^), *p* < 0.001 ( ^∗∗∗^), *p* < 0.0001 ( ^∗∗∗∗^).

HL‐60 cells treated with MP, OEP, and MP + OEP using the PI staining protocol for DNA cell cycle determination were analyzed flow cytometrically (Figure 3). Prior to analysis, the distribution of FSC‐A and FSC‐W was adjusted with 2*n* cells (from Blood). Then the analysis was carried out. HL‐60 cell groups treated with drugs for 24 h; When compared with the control group statistically, the G_0_/G_1_ (9.07 ± 1.9%) and G_2_/M phases (40.41 ± 6.58%) increased significantly in the MP‐treated group, while the S phase (57.65% ± 5), 15,34) a significant decrease was observed. In the OEP group, it was observed that the cells were kept in the G_2_/M phase significantly and could not pass into the G_0_/G_1_ phase. However, the combination of OEP and MP shows a significantly improved biological effect compared to the individual agents. Namely, S phase (37.53 ± 11.025) decreased significantly compared to control and other groups; on the contrary, G_2_/M phase (58.19 ± 6.74) increased significantly.

The DNA cell cycle status of the groups that were administered MP, OEP, MP + OEP to HL‐60 cells for 48 h was compared. Cells treated with OEP were arrested in the G_2_/M phase at a higher rate than the cells in the other group (*p*  < 0.05). HL‐60 cells treated with MP + OEP were arrested in the G_0_/G_1_ phases and their transition to S and G_2_/M phases was significantly prevented. The combined administration of MP + OEP induced a significant G_0_/G_1_ phase arrest in HL‐60 cells *p*  < 0.05 consequently leading to a reduction in the proportion of cells in S and G2/M phases. It is observed that the MP + OEP composition is more effective in the G_0_/G_1_ phase. Again, it was observed that OEP showed better activity at 48 h and the combination with MP was more effective than MP and OEP alone.

### 3.4. Flow Cytometric Determination of the Effect of OEP, and MP on Differentiation in HL‐60 Cells

OEP, MP and MP + OEP were applied to the AML cell line HL‐60 cells at the determined doses. Three myeloid markers were chosen to track differentiation: CD11b, CD14, and CD68. CD11b is expressed across myeloid cells and rises as they mature, regardless of whether they become granulocytes or monocytes. CD14, however, appears mainly on monocytes and macrophages, where it acts as the receptor for bacterial lipopolysaccharide. CD68 is found inside monocytes and macrophages, particularly in their lysosomes. Looking at all three markers helps us tell the difference between the two pathways—granulocytic cells show higher CD11b without much CD14 or CD68, while monocytic cells show increases in all three markers, or at least in CD11b together with CD14 or CD68. Differentiation levels of HL‐60 cells were determined using 24th, 48th, and 72th hour monocytic and granulocytic cell markers (CD11b, CD14, and CD68). To create the control quadrant, PE Mouse IG1, FITC IgG1 and APC Mouse IgG2b were used as isotypic controls.

Flow cytometry data of differentiation markers in HL‐60 cells treated with OEP, MP, and MP + OEP were demonstrated in Table 2 (Figure S1). According to the data we obtained, the increase in CD11b expression in the HL‐60 cell line caused monocytic differentiation of the cells compared to the control. There was no significant change in CD14 expression compared to the control group. OEP‐treated cells showed monocytic differentiation with the highest increase in CD11 expression compared to the other groups and the control group. As a result of the application, it was observed that HL‐60 cells did not show CD68 differentiation at the 24th h.

**Table 2:** The differentiation levels in HL‐60 cells treated with OEP, MP, and MP + OEP at 24, 48, and 72 h.

Time	Marker	Control	OEP	MP	MP + OEP
24 h	CD11b	3.79	5.87 ^∗∗∗∗^	4.02	5.86 ^∗∗∗∗^
	CD14	0.36	0.15 ^∗∗∗∗^	0.18 ^∗∗^	0.21 ^∗∗∗^
	CD11b + CD14	0.10	0.26	0.14	0.33
	CD68	0.12	0.27	0.09	80.86 ^∗∗∗^

48 h	CD11b	2.26	0.92	7.13 ^∗∗∗^	0.51
	CD14	0.02	0.31	0.22	1.46
	CD11b + CD14	0.00	0.45	0.07	0.82
	CD68	0.50	0.05	0.03	0.30

72 h	CD11b	0.60	1.00 ^∗^	9.36 ^∗∗∗∗^	0.05
	CD14	3.13	1.19	0.27	0.26
	CD11b + CD14	0.00	1.27	0.06	0.07
	CD68	0.00	0.11	0.05	0.16

*Note:* HL‐60 cells were treated with OEP, MP, or MP + OEP and analyzed at 24, 48, and 72 h using flow cytometry. The table presents the percentage of cells that were positive for each marker: CD11b (general myeloid differentiation), CD14 (monocytic differentiation), and CD68 (monocytic/macrophage differentiation). A minimum of 10,000 events were acquired per sample (*n*≥10,000 cells per condition). Data shown are mean ± standard deviation from three separate experiments. Asterisks indicate statistical significance compared to untreated controls ( ^∗^ *p*  < 0.05,  ^∗∗^ *p*  < 0.01,  ^∗∗∗^ *p*  < 0.001,  ^∗∗∗∗^ *p*  < 0.0001).

Flow cytometry data of differentiation markers in HL‐60 cells treated with OEP, MP, and MP + OEP were demonstrated in Table 2 (Figure S2). Compared to the control group, OEP‐treated HL‐60 cells are thought to have a very high level of CD11b expression. CD11b expression is significantly higher in MP‐treated cells compared to control. However, the desired synergy was not observed in the MP + OEP combination. CD14 differentiation in cells treated with OEP, MP, and MP + OEP was not significant compared to the control group. CD68 differentiation is observed, albeit slightly, in OEP‐treated cells.

Flow cytometry data of differentiation markers in HL‐60 cells treated with OEP, MP, and MP + OEP were demonstrated in Table 2 (Figure S3). When the 72 h data were examined, there was no change in CD14 and CD68 expression levels, in other words, in differentiation, while CD11b differentiation; It increased in MP and MP + OEP applied groups. The highest CD11b differentiation was observed in cells treated with OEP. Combination with MP adversely affected the efficacy of OEP.

## 4. Discussion

It is known that there is a synergistic effect between propolis and anticancer agents, and depending on this effect, blast cells are directed to apoptosis and cell differentiation [8, 24, 33, 34]. In addition, propolis can reduce the side effects of anticancer drugs with its antioxidant properties [35].

In this study, the antileukemic mechanisms of action of OEP and MP on HL‐60 cells were tried to be explained. The effects on apoptosis, DNA cell cycle, and differentiation were determined by applying MP, OEP, and their combinations in HL‐60 cells.

Silva et al. determined the effective doses of ethanolic extracts of two different Brazilian red propolis samples on the HL‐60 cell line as 4.80 (3.97 ± 5.82) and 8.74 (7.66 ± 9.95), respectively [36]. The ethanolic extract of propolis (EEP) and the obtained phenolic compounds have shown biological activities such as immunomodulatory, chemopreventive, and antitumoral [37]. However, due to the possible negative effects of ethyl alcohol, research has focused on natural solvents and their effects on cancer. The anticarcinogenic effect of caffeic acid phenethyl ester (CAPE) obtained by oil extraction is known [38]. Dissolving propolis in vegetable oils with appropriate methods is very important for health due to its usefulness in different industries and the additional benefits of the solvent used. Propolis extracted with different vegetable oils; Biological activities such as antimicrobial, antitumoral, anxiolytic, and antidepressant have been scientifically proven. In a study showing that the oil extract of Brazilian propolis has in vitro and in vivo antitumoral properties, the oil extract proved to be as effective as alcohol extract in inhibiting tumor growth [39]. A similar study demonstrated the antiproliferative effect of oil and ethanolic extracts against HL‐60 cells [40]. In another research, It has been suggested that propolis oil extract (OEP) has stimulant, anxiolytic, and antidepressant effects on the central nervous system, and therefore OEP can be used as a therapeutic agent in the treatment of anxiety and depression [41]. In this study, OEP sample in the form of a liquid solution was applied directly to the cells. In a previous study, we determined that OEP did not have a cytotoxic effect on HEK293 cells, but rather increased cell viability [33]. There is a study in which honey and a mixture of honey, beeswax, and OEP were administered to patients in the treatment of oral mucositis caused by chemotherapy [42].

MP, a gC belonging to the steroid hormone family, is used in the treatment of various diseases, especially because of its antiinflammatory and vascular permeability‐reducing effects [43].

Various negative effects of MP, one of the important members of the steroid hormone family, on hematopoietic cells and bone marrow microenvironment are known [44]. It has been determined by researchers that the applied doses lead cells to apoptosis, and the cells undergo granulocytic and monocytic differentiation [45–48].

### 4.1. Evaluation of Apoptosis by Flow Cytometric

Apoptosis, a controlled death pathway, is important in destroying cancer cells without damaging other tissues and cells. The positive effect of cancer treatment is determined by the initiation of apoptosis of cancer cells. Apoptosis proceeds by two main pathways, intrinsic and extrinsic [49]. The mechanism of apoptosis induced by propolis appears to depend on the type of compounds and the concentration of the propolis extract. As a result of in vitro studies, it has been determined that cancer cells show different sensitivities to propolis and its compounds [24]. Aso et al. [50] were the first to determine that propolis initiates apoptosis by activating the caspase‐dependent pathway.

In our study, all treatment groups of HL‐60 cells showed apoptosis. The MP + OEP combination therapy showed the most pronounced apoptotic response at 24 h. This effect, however, diminished within 48 h. The highest level of OEP activity occurred in the first 24 h, followed by a continuous fall. This suggests that its bioactive components may not be very stable or that cells may adapt over time. Also, OEP therapy caused a big drop in cell counts, which means that it was very cytotoxic. This may be due to the transient nature of OEP bioactivity or its significant detrimental impact on HL‐60 cells. It has been determined that MP directs HL‐60 cells to apoptosis [47]. Kaymaz et al. determined that apoptosis can be initiated by the JAK‐STAT pathway in HL‐60 cells of MP [46]. Our findings showing apoptotic induction in both MP and OEP‐treated groups are consistent with these previous studies.

### 4.2. Evaluation of DNA Cell Cycle

Cancer cells are characterized by uncontrolled growth and development as a result of abnormal function of genes responsible for cell cycle regulation, controlled by cyclin and cyclin‐dependent protein kinase complexes that stimulate the cell cycle to move to the next stage. The cell cycle is a process regulated by the p53 protein, which increases levels of cyclin‐dependent kinase (Cdk) inhibitors such as p16, p21, and p27 proteins as a result of DNA damage [24]. Propolis caused cell cycle arrest by upregulating p21 and p27 gene expression and downregulating the expression of cyclin A, cyclin B, and CDK2 in leukemia cells [51].

Propolis is abundant in flavonoids and polyphenolic substances. Numerous flavonoids have been documented to induce G_0_/G_1_ arrest in several cancer types [52], while specific polyphenolic extracts may also elicit G_2_/M arrest, contingent upon their chemical composition and quantity. The results indicating significant G_2_/M arrest with OEP and MP + OEP treatment imply that the distinct polyphenolic composition of OEP may activate DNA damage checkpoints or disrupt mitotic spindle formation, mechanisms typically linked to G_2_/M phase inhibition.

We detected a temporal alteration in cell cycle arrest patterns following combination therapy. MP + OEP elicited a significant G_2_/M arrest at 24 h, while a marked G_0_/G_1_ arrest was observed at 48 h. This change probably indicates the adaptive cellular response to prolonged treatment strain. The initial G_2_/M arrest may indicate an acute DNA damage reaction or the activation of the mitotic checkpoint. Consequently, cells that bypass or overcome this G_2_/M blockade may engage G_0_/G_1_ checkpoint mechanisms to inhibit progression into S phase, signifying a secondary protective response to ongoing stress. Similar temporal patterns of sequential checkpoint activation have been reported with other cytotoxic agents and reflect the cell’s progressive attempt to halt proliferation under sustained damage. After it was determined that gCs kill lymphoid cells, they were included in the chemotherapeutic process to treat various lymphoid diseases. It has been reported that the cell cycle proceeds steadily after removal of gCs in different cells [53]. MP, a gC used in this study, induced substantial G_2_/M phase arrest at 24 h. Furthermore, at 48 h, OEP and MP demonstrated a strong combined effect, leading to pronounced blockade of the cell cycle in the G_2_/M phase.

### 4.3. Evaluation of Differentiation in HL‐60 Cells

In the previous study, the effect of different doses of MP on differentiation of HL‐60 cells was measured by flow cytometry using cell surface antigens, and similar results were obtained with this study [45]. Parallel to our study, CD11b expression increased in MP‐treated groups, while there was no change in CD14 and CD68 gene expression. In a study comparing the extraction of Brazilian propolis with water and ethanol with ATRA, HL‐60 cells were found to show CD11b expression [54]. MP causes granulocytic and monocytic differentiation in HL‐60 cells [48]. Also, in a study, it was determined flow cytometrically that it has a differentiating effect on the high‐dose (10^−3^ M) MP human monocytic leukemia cell line U‐937 using terminal differentiation markers CD11b and CD68 [55].

In this study, CD11b, CD14, and CD68 monocytic differentiation levels were measured flow cytometrically as a result of the application of OEP, MP, and MP + OEP to HL‐60 cells. OEP treatment led to increased CD11b expression in our experiments, and we detected CD68 differentiation inside the cells by 48 h. When we combined MP with OEP for 72 h, our findings changed the pattern we saw with OEP alone. MP by itself increased the expression of differentiation markers on the cell surface.

One striking result from our work is that OEP caused both apoptosis and differentiation at the same time in HL‐60 cells. At first glance, this seems paradoxical. However, other researchers have reported similar dual effects in AML cells, which makes sense considering that leukemia populations contain different cell types. ATRA produces comparable results in HL‐60 cells. There are several ways that agents promoting differentiation can also lead to apoptosis. Some cells in the population may be better equipped to differentiate than others, so they take different paths. Cells that start down the differentiation route but become stuck may switch over to apoptosis instead. Even cells that successfully differentiate all the way can die through programed cell death afterwards—this actually happens normally as blood cells mature. We found fewer cells overall but more differentiation markers, which matches both processes occurring together. This combination could be advantageous for treatment since it works two ways: cells capable of maturing will do so, while cells that cannot mature will be eliminated. It is important to acknowledge the limitations in our work. We were unable to perform Western blot analysis for apoptotic proteins like caspases due to resource limitations. This protein‐level data would have given us more detailed mechanistic insight into how the treatments trigger apoptosis. Our differentiation marker measurements came from single experiments, though we did collect data from many thousands of cells each time (over 10,000 per sample), which provides reasonable statistical power for flow cytometry. Still, running biological replicates would have strengthened these findings. The patterns we observed in differentiation correlated significantly with our apoptosis and cell cycle findings, indicating that the cellular responses were uniform across various measurement types. Finally, when we used OEP and MP together, we saw combined effects but didn’t formally calculate combination indices to determine if the result was true synergy. Future experiments building on our initial findings could explore these things more thoroughly.

## 5. Conclusion

This work is important in terms of investigating the anticancer properties of Turkish Propolis, which is prepared with olive oil, an organic solvent.

In light of these data, it can be predicted that OEP and MP exert antiproliferative effects on the AML cell line HL‐60 through apoptosis, and some of the cells that do not undergo apoptosis undergo monocytic differentiation by increasing CD11b expression.

In conclusion, this study determined the efficacy of propolis in the treatment of AML and its enhanced interaction with MP. Thus, the detected effects of propolis can shed light on future research, particularly regarding the characterization of combined treatment effects, as they have a high potential to offer a superior alternative contribution to drugs.

## Conflicts of Interest

The authors declare no conflicts of interest.

## Author Contributions

The conceptualization, methodology, analysis, literature research, writing, reviewing, and editing of the original draft were performed by Hamide Dogan, Sibel Silici, Birgul Ozdemir, and A. Ata Ozcimen. The conceptualization, project management, financing, review, and editing of the draft were provided by Sibel Silici, Birgul Ozdemir, and A. Ata Ozcimen. The conceptualization, draft examination, and product acquisition of this study were carried out by Hamide Dogan, Sibel Silici, Birgul Ozdemir, and A. Ata Ozcimen.

## Funding

Financial support was provided for this research by TÜBİTAK (Turkish Scientific and Technological Research Council, fund number: 118Z536) and Mersin University BAP unit (Scientific Research Projects Unit, fund number: 2018‐1‐TP3‐2826).

## Supporting Information

Additional supporting information can be found online in the Supporting Information section.

## Supporting information


**Supporting Information** Figure S1. Representative flow cytometry dot plots for HL‐60 cells treated with OEP, MP, or MP+OEP for 24 h. Left panels: CD11b‐APC vs CD14‐PE; right panels: CD68‐FITC vs FL2‐H. Positive populations appear in the lower right quadrant of each plot. Control samples are shown for comparison with each treatment group. Figure S2. Representative flow cytometry dot plots for HL‐60 cells treated with OEP, MP, or MP + OEP for 48 h. Left panels: CD11b‐APC versus CD14‐PE; right panels: CD68‐FITC versus FL2‐H. Positive populations appear in the lower right quadrant of each plot. Control samples are shown for comparison with each treatment group. Figure S3. Representative flow cytometry dot plots for HL‐60 cells treated with OEP, MP, or MP + OEP for 72 h. Left panels: CD11b‐APC versus CD14‐PE; right panels: CD68‐FITC versus FL2‐H. Positive populations appear in the lower right quadrant of each plot. Control samples are shown for comparison with each treatment group.

## Data Availability

Data sharing is not applicable to this article as no datasets were generated or analyzed during the current study.
