# Supplementary material for: Apoptosis, Cell Cycle and Differentiation Effects of Propolis Extracted in Olive Oil on HL‐60 AML Cells: A Flow Cytometric Analysis
Source: Anal Cell Pathol (Amst). 2025 Dec 22;2025:7215006. doi: 10.1155/ancp/7215006 (PMC12721472; doi:10.1155/ancp/7215006)
Supplement: Supplementary file 1 — Supporting Information Figure S1. Representative flow cytometry dot plots for HL‐60 cells treated with OEP, MP, or MP+OEP for 24 h. Left panels: CD11b‐APC vs CD14‐PE; right panels: CD68‐FITC vs FL2‐H. Positive populations appear in the lower right quadrant of each plot. Control samples are shown for comparison with each treatment group. Figure S2. Representative flow cytometry dot plots for HL‐60 cells treated with OEP, MP, or MP + OEP for 48 h. Left panels: CD11b‐APC versus CD14‐PE; right panels: CD68‐FITC versus FL2‐H. Positive populations appear in the lower right quadrant of each plot. Control samples are shown for comparison with each treatment group. Figure S3. Representative flow cytometry dot plots for HL‐60 cells treated with OEP, MP, or MP + OEP for 72 h. Left panels: CD11b‐APC versus CD14‐PE; right panels: CD68‐FITC versus FL2‐H. Positive populations appear in the lower right quadrant of each plot. Control samples are shown for comparison with each treatment group. [file ANCP-2025-7215006-s001.docx]

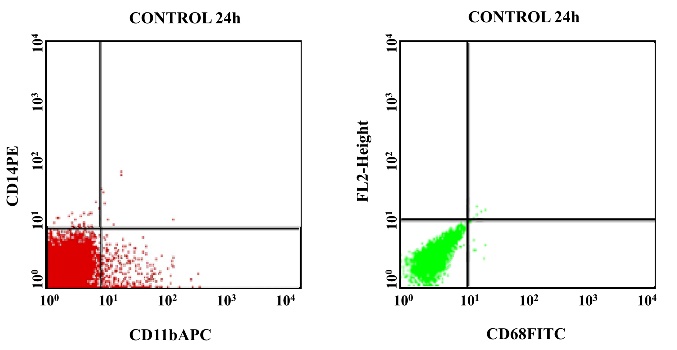

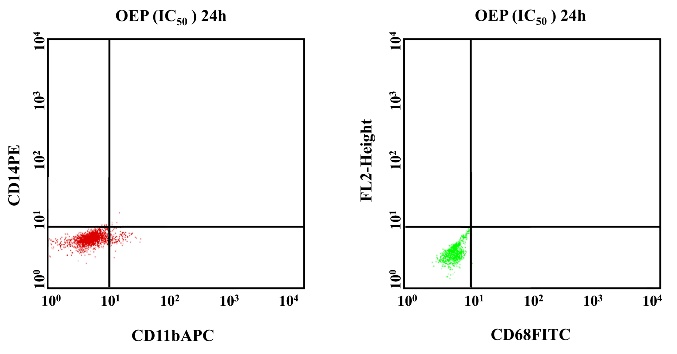


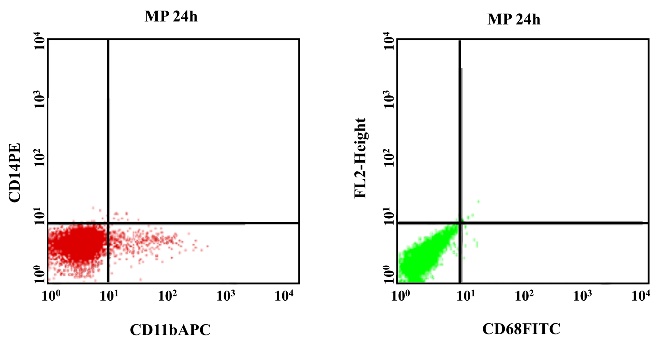

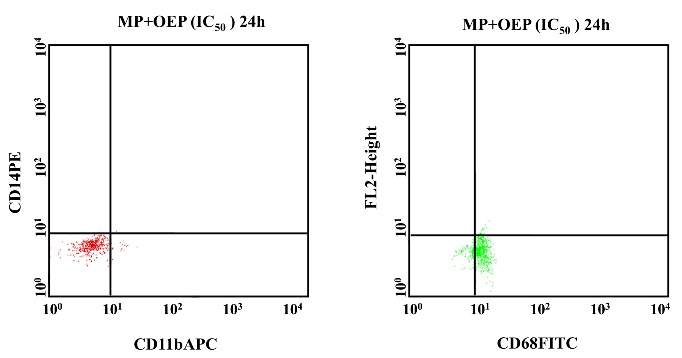


**Figure S1.** Representative flow cytometry dot plots for HL-60 cells treated with OEP, MP, or MP+OEP for 24 hours. Left panels: CD11b-APC vs CD14-PE; right panels: CD68-FITC vs FL2-H. Positive populations appear in the lower right quadrant of each plot. Control samples are shown for comparison with each treatment group.


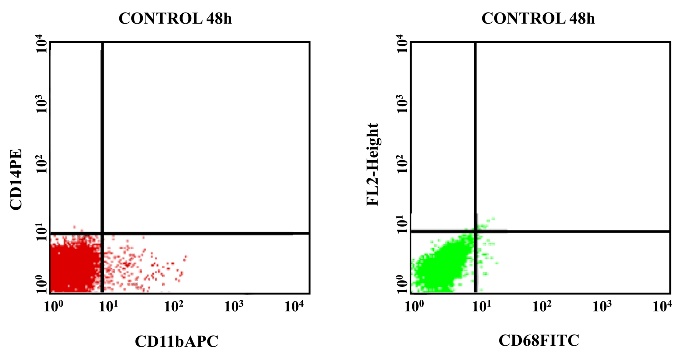

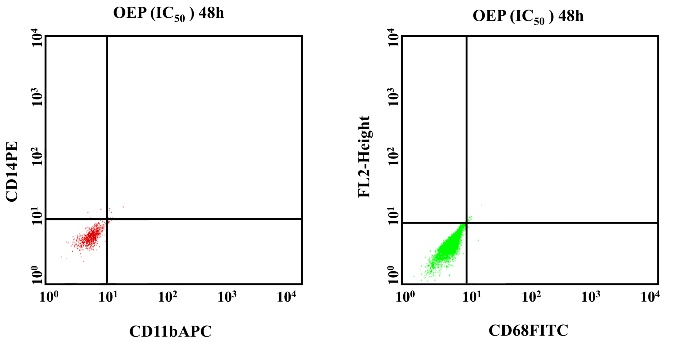


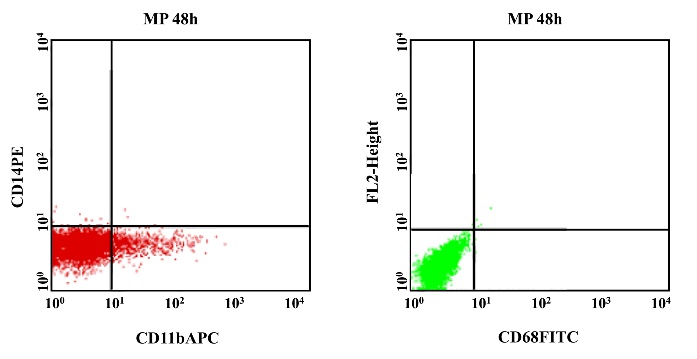

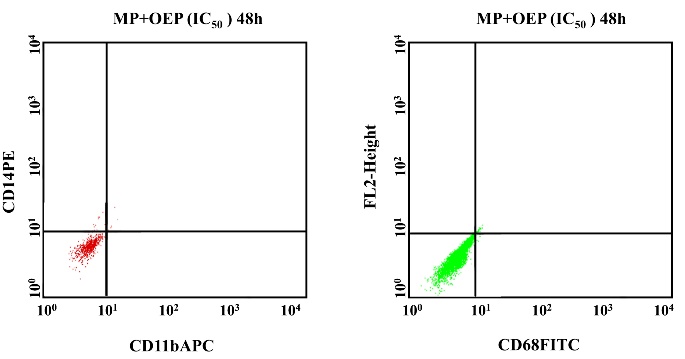


**Figure S2**. Representative flow cytometry dot plots for HL-60 cells treated with OEP, MP, or MP+OEP for 48 hours. Left panels: CD11b-APC vs CD14-PE; right panels: CD68-FITC vs FL2-H. Positive populations appear in the lower right quadrant of each plot. Control samples are shown for comparison with each treatment group.


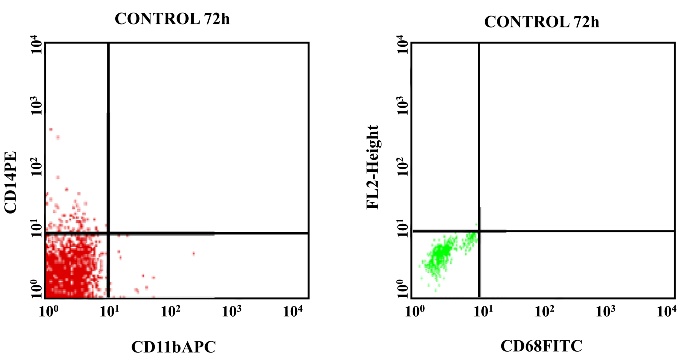

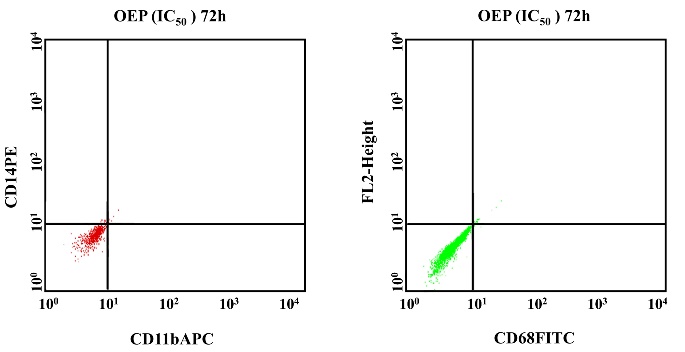


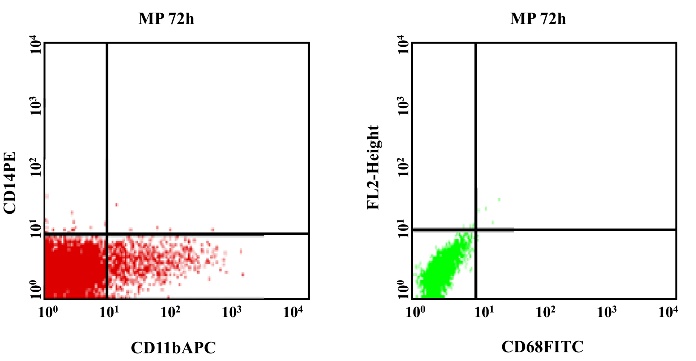

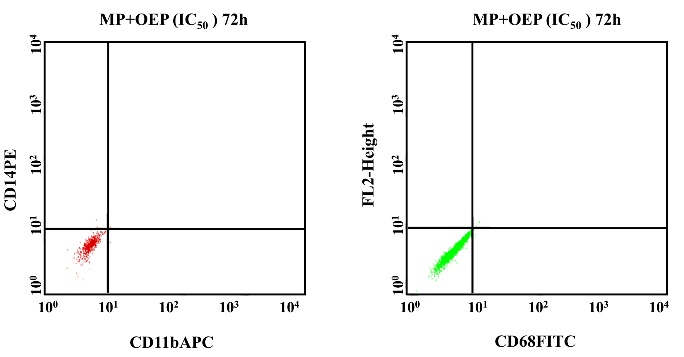


**Figure S3.** Representative flow cytometry dot plots for HL-60 cells treated with OEP, MP, or MP+OEP for 72 hours. Left panels: CD11b-APC vs CD14-PE; right panels: CD68-FITC vs FL2-H. Positive populations appear in the lower right quadrant of each plot. Control samples are shown for comparison with each treatment group.
